# Septal urocortin 3 modulates stress-coping behaviour but not hypothalamic-pituitary-adrenal axis activity during forced swimming

**DOI:** 10.1186/1471-2210-11-S2-A43

**Published:** 2011-09-05

**Authors:** Karl Ebner, Alesja Rjabokon, Nicolas Singewald

**Affiliations:** 1 Department of Pharmacology and Toxicology, Institute of Pharmacy, and Center for Molecular Biosciences Innsbruck (CMBI), University of Innsbruck, 6020 Innsbruck, Austria

## Background

The lateral septum (LS) has been shown to play an important role in the generation and modulation of behavioural and neuroendocrine stress responses. However, the exact neurochemical mechanisms mediating these effects are not well studied so far. Several lines of evidence suggest a robust contribution of septal urocortin 3 (UCN3) and its preferred receptor, the corticotropin-releasing factor type 2 (CRF2) receptor in mediating these effects. Therefore, the aim of the present study was to examine the role of septal CRF2 receptors in neuroendocrine and behavioural stress responses.

## Methods

Male Sprague-Dawley rats implanted with a jugular venous catheter and a microinjection cannula aimed at the LS received bilateral injections of either UCN3, the selective CRF2 receptor antagonist anti-sauvagine-30 (ASV-30) or vehicle, and were exposed to forced swim stress.

## Results

Our data show that intraseptal UCN3 infusion reduced active coping and increased immobility during the forced swim exposure as UCN3 treated animals showed less struggling and swimming behaviour and increased floating behaviour compared to controls. Conversely, the administration of ASV-30 had the opposite effects, an increase in struggling and swimming and a reduction in floating. In contrast to the behavioural stress response, the administration of UCN3 or ASV-30 directly into the LS had no effect on either basal or stress-induced increase of plasma ACTH levels, indicating that septal CRF2 receptors are not involved in hypothalamic-pituitary-adrenal (HPA) axis regulation.

## Conclusions

Taken together, our data suggest that CRF2 receptors in the LS are critically involved in the regulation of behavioural stress reactivity but not in HPA axis regulation.

